# Prescription Department

**Published:** 1891-10

**Authors:** 


Unna recommends the following liniment for Eczema of Anus and Scrotum (L'Union Med., July 2d):

R Zinci oxidi, oz. j.
Iodoform, dr. iss.
Olei lini, oz j.
Aquae calcis, f oz. j, M.

For Stomatitis in children, a writer in Province Medicale (quoted in Boston Medical and Surgical Journal, July 30th) suggests the following treatment:

R Cocain. hydrochlorat., gr. iss.
Sodii chlorid., gr. xv.
Glycerine,
Aquae, aa f dr. ijss. M.

Brush gums with camel's hair brush. Also use freely spray of boracic acid, and give potassium brom. internally.

A new treatment of Herpes Zoster is the following (Semaine Med., 1891, in Merek's Bulletin, June, 1891):

R Extract gelsemii, gr. xxx.
Sodi sulphocarbolat, dr. j.
Aquae distillat., f oz. iij. M.

Sig. A teaspoonful every two hours.

At the same time, five drops of tincture of belladonna are administered every two hours until slight dryness of the pharynx is experienced. Effects of the gelsemium should be carefully watched.

R Plumbi acetat.,
Alum pulv., aa dr. j.
Aquae distillat., f oz. iv. M.

Sig. Use externally.

Compresses moistened with this solution are applied to the affected parts and renewed every two hours. Pain is said to disappear within a few hours and the disease to be considerably shortened by this treatment.

Dr. Brubaker (Medical World) recommends the following for Spasmodic Cough:

R Acid hydrocyan. dilut., f dr. j.
Tinct. sanguinariae, f dr. iv.
Syrup senegae f dr. ss.
Syrup tolu, f dr. ij.
Aquae laurocerasi, q. s. ad f oz viij

M. Sig. F dr. j. t. d.

In Amenorrhoea, a writer in Revue Med.-chir. des Mal. des. Femmes prescribes—

R Bichloride of mercury, 3 grains.

Arsenite of sodium, 3 grains.

Sulphate of strychnine, 1½ grains.

Carbonate of potass. } each 45 grs.

Sulphate of iron, }

M. Make into 60 pills and give one pill after each meal.

A favorite prescription of the outdoor medical department of the Jefferson Medical College Hospital, in cases of difficult digestion accompanied by constipation and flatulency, is the following (Medical and Surgical Reporter, August 15):

R Tinct. nucis vomicae.
Tinct. belladonna.
Tinct. physostigmatis, aa f dr. ij.
Ext. cascarae fluid, f dr. vi.
Ext. erythroxyli fluid, q. s. f oz. iij.
M, Sig. A teaspoonful after each meal.

The following is a dispensary prescription for Diarrhoea (Buffalo Medical and Surgical Journal, August, 1891):

R Salol, dr. ij.
Bismuth subnitrat, dr. iv.
Misturae cretae, oz. f iij. M.
Sig. A teaspoonful every two hours.

As a gargle to prevent Dental Caries, New Remedies recommends the following:

R Tanniu, 5 gm.
Tincture of iodine.
Tincture of myrrh, aa 2½ gm.
Iodide of potassium, 1 gm.
Rose water, 180 gm. M.

Sig. A teaspoonful in a glass of water as a gargle every morning.

For Fetid Breath, F. Shoen, (Internat. Klin. Rundsch.), suggests the following mouth-wash:

R Saccharin.
Sodii bicarb., aa gr. xv.
Acid sallicylic, dr. j.
Alcohol, f dr. ij-oz. vj. M.

Sig. A few drops in a glass of water.

